# Robust and semantic needle detection in 3D ultrasound using orthogonal-plane convolutional neural networks

**DOI:** 10.1007/s11548-018-1798-3

**Published:** 2018-05-31

**Authors:** Arash Pourtaherian, Farhad Ghazvinian Zanjani, Svitlana Zinger, Nenad Mihajlovic, Gary C. Ng, Hendrikus H. M. Korsten, Peter H. N. de With

**Affiliations:** 1 Eindhoven University of Technology, 5612 AJ Eindhoven, The Netherlands; 2 Philips Research Eindhoven, 5656 AE Eindhoven, The Netherlands; 3 Philips Healthcare, Bothell, WA 98021 USA; 4 Catharina Hospital Eindhoven, 5623 EJ Eindhoven, The Netherlands

**Keywords:** Needle detection, 3D ultrasound, Convolutional neural networks

## Abstract

**Purpose:**

During needle interventions, successful automated detection of the needle immediately after insertion is necessary to allow the physician identify and correct any misalignment of the needle and the target at early stages, which reduces needle passes and improves health outcomes.

**Methods:**

We present a novel approach to localize partially inserted needles in 3D ultrasound volume with high precision using convolutional neural networks. We propose two methods based on patch classification and semantic segmentation of the needle from orthogonal 2D cross-sections extracted from the volume. For patch classification, each voxel is classified from locally extracted raw data of three orthogonal planes centered on it. We propose a bootstrap resampling approach to enhance the training in our highly imbalanced data. For semantic segmentation, parts of a needle are detected in cross-sections perpendicular to the lateral and elevational axes. We propose to exploit the structural information in the data with a novel thick-slice processing approach for efficient modeling of the context.

**Results:**

Our introduced methods successfully detect 17 and 22 G needles with a single trained network, showing a robust generalized approach. Extensive ex-vivo evaluations on datasets of chicken breast and porcine leg show 80 and 84% *F*1-scores, respectively. Furthermore, very short needles are detected with tip localization errors of less than 0.7 mm for lengths of only 5 and 10 mm at 0.2 and 0.36 mm voxel sizes, respectively.

**Conclusion:**

Our method is able to accurately detect even very short needles, ensuring that the needle and its tip are maximally visible in the visualized plane during the entire intervention, thereby eliminating the need for advanced bi-manual coordination of the needle and transducer.

## Introduction

Ultrasound (US) imaging is broadly used to visualize and guide the interventions that involve percutaneous advancing of a needle to a target inside the patients’ body. However, for a typical 2D US system, bi-manual coordination of the needle and US transducer is challenging, as the limited US field of view obscures the visualization of the complete needle and an inadequate view leads to an erroneous placement of the needle tip. Therefore, while advancing the needle, continuous manipulation of the transducer is necessary to search for the needle in the imaging data for the best needle plane visualization. As an alternative, 3D US transducers with an image-based needle-tracking system can overcome these limitations and minimize the manual coordination, while preserving the use of a conventional needle, signal generation and transducers [12]. In such a system, the needle is conveniently placed in the larger 3D US field of view and the processing unit automatically localizes and visualizes the entire needle. Therefore, the manual skills are significantly simplified when the entire needle is visible in the visualized plane, after the needle is advanced or the transducer is moved.

Several image-based needle localization techniques have been proposed based on maximizing the intensity over parallel projections [1]. Due to the complexity of realistic data, methods that solely rely on the brightness of the needle are not robust for localizing thin objects in a cluttered background. Therefore, information regarding the line-like structure of a needle is used by Hessian-based line filtering methods [21]. Although shown to be limited in localization accuracy [12], they can be beneficial for reducing the imaging artifacts. Other techniques involve exploiting the intensity changes caused by needle movement to track the needle in the US data [2]. Nevertheless, large movements of the transducer or the patient will increase the difficulty of motion-based tracking and therefore, we aim at repeated detection in static 3D volumes. When realizing the real-time operation, tracking of the needle is implemented by repeated detection with sufficient time resolution. This will result in detection per volume in a 4D US sequence, which allows for arbitrary inter-volume movements.

More recently, attenuation of the US signal due to energy loss beyond the US beam incident with the needle, is used to detect the position of the needle [8, 11]. However, signal loss due to the presence of other attenuating structures may degrade the accuracy of estimation and must be explicitly handled. Alternatively, supervised needle-voxel classifiers that employ the needle shape and its brightness have shown to be superior to the traditional methods [12]. Nevertheless, as the needle is assumed to be already inserted in the volume up to a considerable length, they typically do not achieve high detection precision and therefore cannot localize the needle when it is partially inserted in the volume. Moreover, when the target structure is deep, the degraded resolution and possible needle deflections further complicate the interpretation of data and reduce voxel classification performance, which should be addressed by better modeling of both local and contextual information.

In our recently published work in training convolutional neural networks (CNN), substantial improvement has been shown to the detection accuracy of needle voxels in 3D US data [10]. Although this method was shown to achieve high-performance results for ex vivo data acquired from linear-array transducers, the choice of patch classification in this framework can be further improved for US data segmentation. In US imaging using sector, curved and phased-array transducers, the insonification angle of the US beams is changed throughout the volume, which creates varying angles with different parts of the needle. Therefore, the needle can be partially invisible, due to the lack of received US reflections from parts of the needle. The missed data enforces a trade-off between patch sizes for richer needle context information and localization accuracy. Larger patches require more max-pooling layers that reduce the localization accuracy, while small patches allow the network to infer from only parts of the needle.

**Fig. 1:**
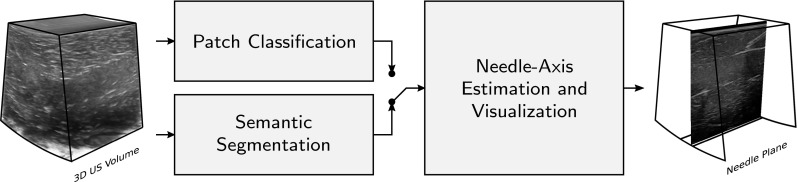
Block diagram of our proposed framework for needle detection in 3D US data

As an alternative to patch training, semantic segmentation methods can generate dense prediction maps by omitting the use of fully connected layers. Examples of such networks are fully convolutional networks (FCN) [7, 16], and context modeling by employing atrous convolutions [3, 24]. Although integrating atrous (or dilated) convolutions in the deep layers of the network increases the field of view while preserving the spatial dimensions, applying convolutions in a large number of high-resolution feature maps is computationally expensive. However, original FCN architectures can simultaneously exploit the global and local information in the data and remain more memory efficient by introducing skip connections from higher-resolution feature maps to the deconvolutional layers. Initial attempts of applying these networks on US data are presented for fetal heart segmentation in 2D US [19]. Further improvement is shown for segmentation of fetus, gestational sac, and placenta in 3D US volumes by integrating the sequential information [23]. The drawback of using such 3D+time models are, however, the exponentially increased computational complexity of the 3D convolution operations, a very large dataset is required for training the increased number of network parameters, and the sequential modeling will be suboptimal in the early timesteps after large movements of the transducer or subject.

In this paper, we build upon our recent contribution using CNN [10] and extend it to create semantic FCN models. We modify and extend this architecture such that it models 3D needle context information and achieves high needle segmentation precision at a low false negative rate. We propose a novel multi-view thick-sliced FCN model for an efficient modeling of 3D context information. Therefore, the system will successfully perform needle localization both in cases of invisible needle parts and when only a short part of the needle is inserted into the patient’s body, yielding an early correction of inaccurate insertions. The needle is then visualized in an intuitive manner, eliminating the need for advanced manual coordination of the transducer. The main contributions of this paper are: (1) a novel approach for segmentation and localization of partially inserted and partly invisible needles in 3D US data using CNN models, (2) an original update strategy for CNN parameters using most-aggressive non-needle samples, which significantly improves the performance on highly imbalanced datasets, (3) a novel method for modeling of 3D US context information using 2.5D (thick slice) data, which enables an accurate training of the network using limited training samples, and (4) extensive evaluation of the proposed methods on two types of ex-vivo data giving a very high average of 81.4% precision at 88.8% recall rate.

## Methods

The block diagram of our proposed framework consists of three main stages as depicted in Fig. 1. In this study, we introduce two different approaches for segmentation of needle voxels in 3D US volumes, i.e., classification of extracted patches in the data using their triplanar orthogonal views (“Patch classification” subsection), and end-to-end dense segmentation of needle voxels in multi-view thick slices (“Semantic segmentation” subsection). The segmentation results are then used to fit a predefined model of the needle and extract the cross-section containing the entire needle and the tip (“Needle axis estimation and visualization” subsection). For clarity, we emphasize here that we detect the plane where the needle and its tip are maximally visible, but do not explicitly detect the needle tip. This localization processing is done for every data volume individually. For a 3D+time US sequence, this would effectively mean repeated detection for every volume or image.

### Patch classification

**Fig. 2:**
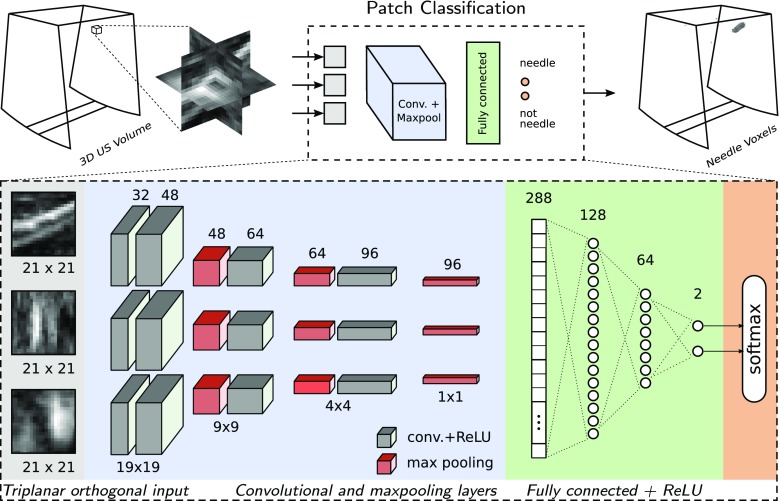
Block diagram of the patch classification approach using CNN

The block diagram of the proposed patch classification technique is shown in Fig. 2. A CNN model is trained to robustly classify the needle voxels in the 3D US volumes from other echogenic structures, such as bones and muscular tissues. Our voxel classification network predicts the label of each voxel from the raw voxel values of local proximity. In a 3D volume, this local neighborhood can simply be a 2D cross-section in any orientation, multiple cross-sections, or a 3D patch. Here, we use three orthogonal cross-sections centered at the reference voxel, which is a compromise with respect to the complexity of the network. The size of triplanar cross-sections is chosen based on the diameter of a typical needle (0.7–1.5 mm), voxel size, and spatial resolution of the transducer, to contain sufficient context information. We extract triplanar cross-sections of $21 \times 21$ pixels ($4.2 \times 4.2$ mm), which provides sufficient global shape information and still remains spatially accurate. For low-frequency transducers, more context is required for a discriminative modeling, as the structure details of a needle will be distorted at low spatial resolutions.

*CNN architecture* For our experiments, we evaluate two CNN architectures based on shared convolutional (ShareCNN) and independent convolutional (IndepCNN) filters. In ShareCNN, a single convolutional filter bank is trained for the three input planes to have the same set of filters for all the planes. In IndepCNN, three sets of filter banks are trained independently, each to be convolved with one of the three planes. As depicted in Fig. 2, both architectures consist of four convolutional layers having 32, 48, 64 and 96 filters of $3 \times 3$ kernel size, three fully connected layers having 128, 64 and 2 neurons, and one softmax layer. According to the given number of filters, ShareCNN and IndepCNN architectures have 2160 and 6480 parameters in their convolutional layers, respectively. In both architectures, extracted feature maps after the last convolutional layer are concatenated prior to the fully connected layers [14].

**Fig. 3:**
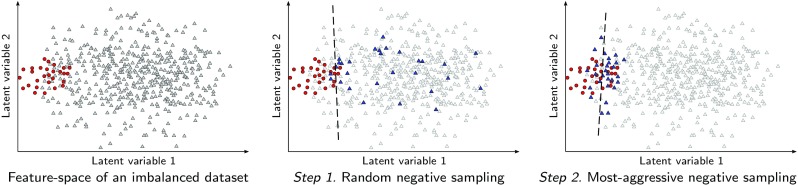
An example of the iterative sampling strategy to increase the precision of the network. The red circles represent the positive data points, gray and blue triangles are the negative and sampled data points, respectively and the dashed line represents the decision boundary of a classifier

*CNN training* Our dataset is significantly imbalanced due to the small size of a needle compared to the full volume, i.e., approximately only 1 voxel out of 3000 voxels in a volume belongs to the needle. This is common in representation of an instrument in 3D US volumes. Therefore, in order to avoid a prediction bias toward the majority class, we downsample the negative training data to match the number of needle samples. For an informed sampling of the negative (non-needle) set, we propose an iterative scheme based on bootstrapping [15] to achieve the maximum precision. In the first step, we train our network with uniformly sampled needle and non-needle patches. Training patches are rotated arbitrarily by 90$^{\circ }$ steps around the axial axis to improve the orientation invariance. The trained network then classifies the same training set for validation. Finally, misclassified false positives are harvested as the most-aggressive non-needle voxels, which are used to update the network. Figure 3 shows how the iterative and informed sampling can increase the precision of the network. It is worth mentioning that commonly used methods for imbalanced data, like weighted loss function, do not necessarily improve precision. For example, the majority of our negative set consists of “easy” samples that can be classified beyond the model’s margin and will influence the loss function in their favor.

The CNN parameters are trained using stochastic gradient descent (SGD) and the categorical cross-entropy cost function. All activation functions are chosen to be rectified linear units (ReLU) [5]. Furthermore, for optimization of the network weights, we divide the learning rate by the exponentially weighted average of recent gradients (RMSProp) [20]. Initial learning rates are chosen to be $10^{-4}$ and $10^{-5}$ for train and update iterations, respectively. In order to prevent overfitting, we implement the dropout approach [18] with a probability of 0.5 in the first two fully connected layers. The trained network computes a label per voxel indicating whether it belongs to the needle or not.

### Semantic segmentation

As discussed in “Introduction” section, semantic segmentation of a needle using FCN architectures are more interesting than patch classification as the context information is modeled, while the spatial dimensions are preserved. Furthermore, in contrast to the patch-based methods, where redundant processing of voxels is inevitable, FCN models are more computationally efficient as they exploit the one-time extracted features to simultaneously label all the data points using deconvolutional networks.

**Fig. 4:**
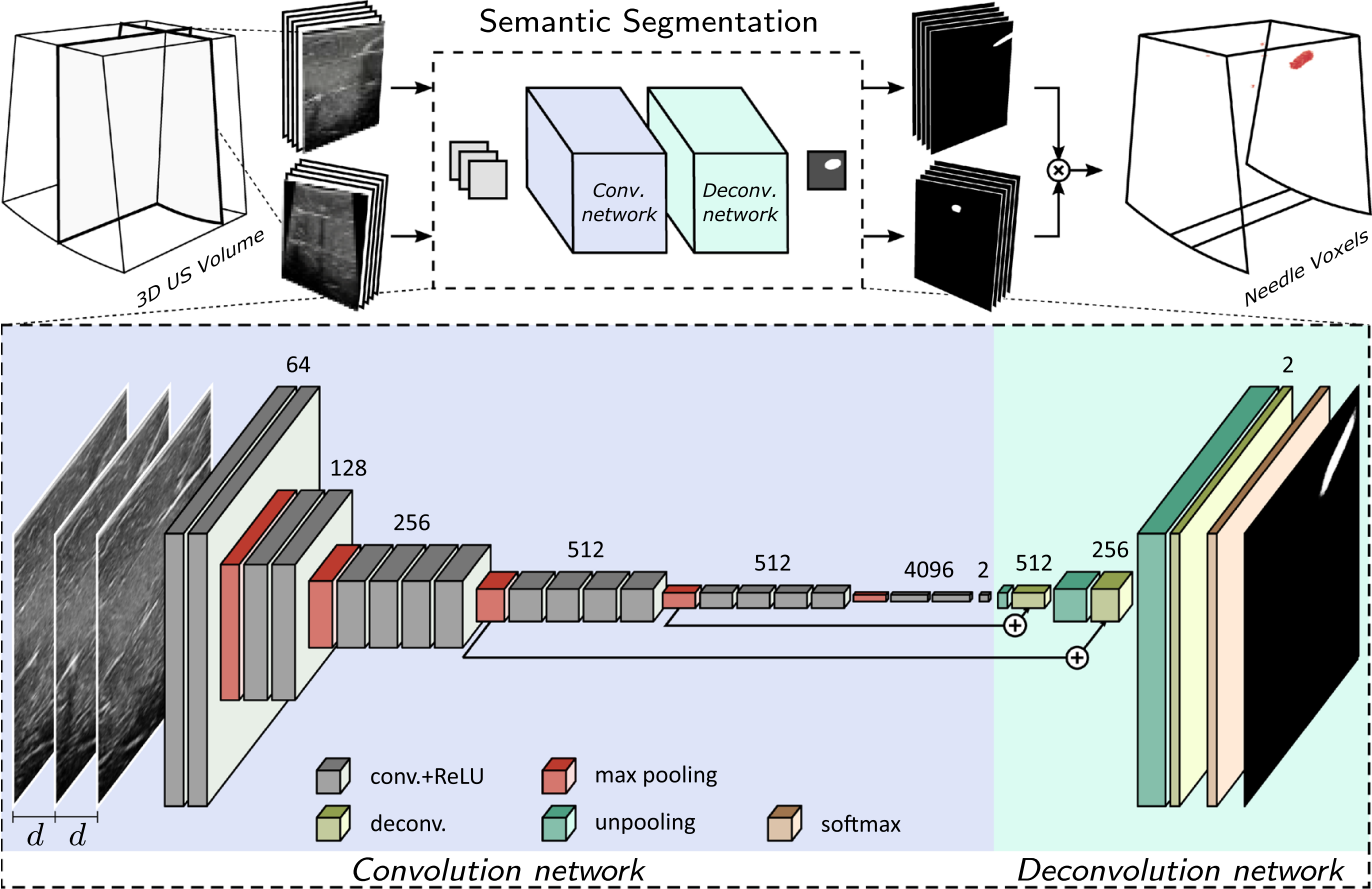
Block diagram of the semantic segmentation approach using FCN

Figure 4 shows the architecture of the proposed semantic needle segmentation technique in 3D US volumes. Our method is based on decomposing the 3D volume into 2D cross-sections for labeling the needle parts and reconstructing the 3D needle labels from the multiple views. Therefore in our approach, the number of parameters in the convolution kernels decreases exponentially compared to the 3D kernels, and consequently, the network requires fewer training samples and executes faster. We will now present our strategy for selecting the cross-sections to be processed.

The 2D cross-sections are selected in multiple directions and perpendicular to the transducer with the step size equal to the voxel size. Since in a 3D US volume, the needle can enter the field of view from either the lateral or elevational directions, we consider cross-sections perpendicular to these axes. The segmentation outcome of each cross-section is mapped onto its corresponding position in 3D. Afterward, the resulting probability volume from the two directions is combined together using multiplicative averaging to create the final labeling outcome in 3D.

In order to exploit the 3D structural information in our model, instead of only using 2D planar data, we opt for processing the consecutive cross-sections before and after the processing plane as additional inputs to the network. In this study, we add two additional cross-sections and evaluate several spacing gaps *d*, between them. Therefore, as shown in Fig. 4, a 3-channel input to the network is formed from the 2.5D (thick slice) US data at a specific position, which is used to create a 2D segmentation map of the processing cross-section.

*FCN architecture* Figure 4 depicts the FCN architecture used in our system comprising two stages of convolution and deconvolution networks. Inspired by ShareCNN, we use shared convolution filters (ShareFCN) for both lateral and elevational planes. The convolution network is identical to the design of the VGG very deep 19 layer CNN [17]. The deconvolution network consists of three unpooling masks of 2, 2 and 8 pixels, respective convolution layers having 512, 256 and 2 filters with $3 \times 3$ kernel size, and one softmax layer. Therefore, the receptive field of the network is equal to a window of $96 \times 96$ pixels, which is equivalent to approximately $19.2 \times 19.2$ mm for higher-resolution VL13-5 transducer and $34.5 \times 34.5$ mm for lower-resolution X6-1 transducer, achieving a large context modeling at the same inference resolution of the input data. Convolution layers are stacked together followed by an activation function. As discussed, the network takes a 3-channel 2D input and the output layer indicates the class-assignment probability for each pixel in the processing cross-section.

*FCN training* The training set consists of 3-channel cross-sections extracted with a gap of *d* mm in both elevational and lateral directions. The training volumes are augmented by 10 arbitrary rotations around the axial (*z*) axis prior to extraction of the cross-sections. Therefore, several views of the needle are used to train the network, including in-plane, out-of-plane and cross-sections with partial visibility of the needle. Similar to our approach presented in “Patch classification” subsection, we downsample the negative training data, which are the sections that do not contain the needle, to match the number of cross-sections from the needle. However, since the initial training samples are not highly imbalanced, we do not perform bootstrapping for training the FCN parameters.

We trained the network parameters using SGD update with a batch size of one sample and softmax cross-entropy cost function. The learning rate is adaptively computed using the ADAM optimization method [6] with an initial learning rate equal to 1e−4. Furthermore, dropout layers with a probability of 0.85 are added to the layer numbers 17 and 18 of the convolution network.

### Needle axis estimation and visualization

In order to robustly detect the instrument axis in the presence of outliers, we fit a model of the needle to the detected voxels using the RANSAC algorithm [4]. The needle model can be represented by a straight cylinder having a fixed diameter. In cases of large instrument deflection, the model can be adapted to define a parabolic segment, as shown in [9]. Using the RANSAC algorithm, the cylindrical model that contains the highest number of voxels is chosen to be the estimated needle. As the experimented needle diameters are less than 2 mm, we set the cylindrical model diameter to be approximately 2 mm.

After successful detection of needle axis, the 2D cross-section of the volume is visualized that contains the plane with the entire needle with maximum visibility, which is also perpendicular to coronal (*xy*) planes. This cross-section is the in-plane view of the needle that is very intuitive for physicians to interpret. This ensures that while advancing the needle, the entire instrument is visualized as much as it is visible and any misalignment of the needle and target is corrected without maneuvering the transducer.

### Implementation details

Our Python implementations of the proposed patch classification and semantic segmentation methods take on average 74 and 0.5 µs for each voxel, respectively (1180 and 15 ms for each 2D cross-section) on a standard PC with a GeForce GTX TITAN X GPU. Therefore, when implementing a full scan to process all voxels and cross-sections in the volume, patch classification executes in 4–5 min, whereas semantic segmentation takes only 2–3 s. Nevertheless, further optimization is possible using conventional techniques such as a coarse-fine search strategy with a hierarchical grid to achieve real-time performance. Furthermore, the execution time of RANSAC model fitting is negligible, as the expected number of outliers is very small.

The required computational power for realization of our proposed methods is expected to be widely available on high-end ultrasound devices that benefit from parallel computing platforms, such as a GPU. However, for implementation in mid-range and portable systems, more efficient and compressed architectures should be investigated. Still, ever-increasing computational capacity of mobile processors, as well as fast development and availability of on-board embedded units with pre-programmed convolutional modules will make such computer-aided applications more affordable and readily accessible to the majority of ultrasound devices.

**Table 1:** Specifications and experimental settings of 3D US volumes used for evaluation

Tissue type / transducer	Needle type and diameter	Experimental settings			Voxel size (mm)
		# of vols.	Maximum length (mm)	Steepness angles	
Chicken breast/ VL13-5$^{\hbox {a}}$	17 G (1.47 mm)	10	30	$10^{\circ }{-}30^{\circ }$	0.20
	22 G (0.72 mm)	10	30	$5^{\circ }{-}50^{\circ }$	0.20
Porcine leg/ X6-1$^{\hbox {a}}$	17 G (1.47 mm)	10	45	$55^{\circ }{-}80^{\circ }$	0.36
	22 G (0.72 mm)	10	35	$20^{\circ }{-}65^{\circ }$	0.36

$^{\hbox {a}}$Available from Philips Healthcare, Bothell, WA, USA

## Experimental results

The evaluation dataset consists of four types of ex-vivo US data acquired from chicken breast and porcine leg using a VL13-5 transducer (motorized linear-array) and a X6-1 transducer (phased-array). Our experiments with two types of transducers and tissue types investigate the robustness of the proposed methods in various acquisition settings and conditions. Properties and specifications of our dataset are summarized in Table 1. Each volume from VL13-5 transducer contains on average $174 \times 189 \times 188$ voxels (lat. $\times$ ax. $\times$ elev.), at 0.2 mm/voxel and from X6-1 transducer contains $452 \times 280 \times 292$ voxels, at approximately 0.36 mm/voxel. Ground-truth data is created by manually annotating the voxels belonging to the needle in each volume. Testing evaluation is performed based on five-fold cross-validation separately for each transducer across its 20 ex-vivo 3D US volumes. For each fold, we use 4 subsets for training and 1 subset for testing, to make the training and testing data completely distinct.

### Patch classification

**Fig. 5:**
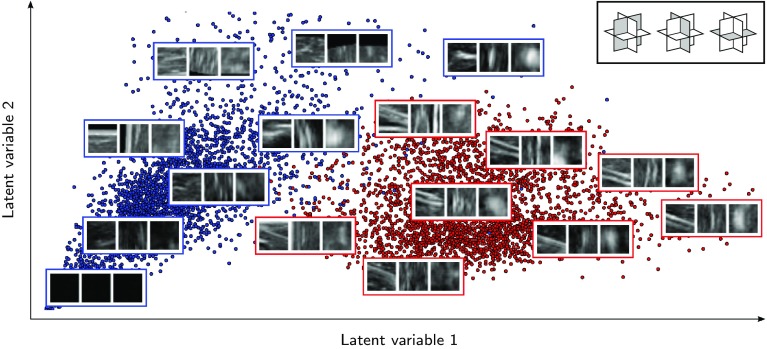
Multi-dimensional projection of voxels in the test set using the *t*-SNE algorithm. Red and blue points represent needle and non-needle voxels, respectively

We use the dataset from chicken breast to evaluate the performance of the proposed patch classification method. Capability of the network to transform the input space to meaningful features is visualized using a multi-dimensional scaling that projects the representation of feature space onto a 2D image. For this purpose, we applied *t*-distributed Stochastic Neighbor Embedding (*t*-SNE) [22] to the first fully connected layer of the network. The result of the multi-dimensional projection of the test set in one of the folds is depicted in Fig. 5, where close points have similar characteristics in the feature space. As shown, the two clusters are clearly separated based on the features learned by the network.

Performance of our proposed methods is evaluated in the full volumes and the results are shown in Table 2, listing voxel-level recall, precision, specificity and *F*1-score. Recall is the sensitivity of detection and is defined as the number of correctly detected needle voxels divided by the number of voxels belonging to the needle. Precision or the positive predictive value is defined as the number of correctly detected needle voxels divided by the total number of detected needle voxels. Specificity is defined as the number of voxels that are correctly detected as non-needle divided by the number of voxels that are not part of the needle. Finally, *F*1-score is calculated as the harmonic mean between the voxel-based recall and precision and is used to measure the similarity between the system detections and the ground-truth labels.

Furthermore, we compare the results with the approach of [13], which is based on supervised classification of voxels from their responses to hand-crafted Gabor wavelets. As shown, both shared CNN and independent CNN architectures outperform the Gabor features yielding a 25% improvement on *F*1-score. Furthermore, ShareCNN achieves higher precision than IndepCNN at approximately similar recall rate. The degraded performance of IndepCNN can be explained by the large increase in the number of network parameters in our small-sized data.

**Table 2:** Average voxel classification performances in the full volumes of chicken breast (%)

Method	Recall	Precision	Specificity	*F*1-score
Gabor transformation$^{\hbox {a}}$ [13]	47.1	48.2	–	53.7
ShareCNN$^{\hbox {b}}$	76.3 ± 5.8	83.2 ± 5.6	99.98 ± 4e−5	78.5 ± 5.3
IndepCNN$^{\hbox {b}}$	78.4 ± 5.3	64.7 ± 4.8	99.97 ± 6e−5	66.1 ± 4.9

$^{\hbox {a}}$Two models trained separately for each needle (averaged)

$^{\hbox {b}}$Single model trained directly for both needles

**Fig. 6:**
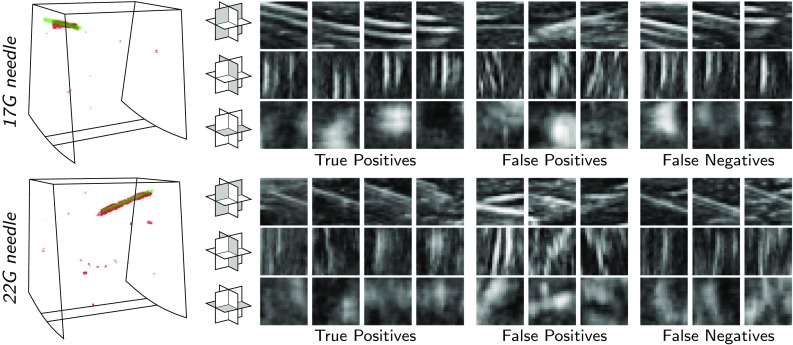
Examples of classification results for 17 and 22 G needles. (Left) Detected needle voxels in 3D volumes shown in red and ground-truth voxels in green. (Right) Triplanar orthogonal patches classified as true positive, false positive, and false negative

Figure 6 shows examples of the classification results for 17 and 22 G needles. As shown in the left column, detected needle voxels correctly overlap the ground-truth voxels, which results in a good detection accuracy. Furthermore, example patches from true and false positives are visualized, which show a very high local similarity. Most of the false negative patches belong to the regions with other nearby echogenic structures, which distorts the appearance of the needle.

### Semantic segmentation

We evaluate the performance of our proposed semantic segmentation method on both datasets from chicken breast and porcine leg. As shown in Table 1, the data of porcine leg are acquired using a phased-array transducer, in which the needle appearance will be more inconsistent due to the varied reflection angles of the backscattered beams.

#### Data representation in 2.5D

As discussed in “Semantic segmentation” section, we use a 3-channel input to the FCN network for better modeling of the 3D structures from 2.5D (thick slice) US data. The three channels consist of parallel cross-sections having *d* mm gap between them. In this section, we investigate the contribution of our multi-slicing approach for increasing the segmentation accuracy of individual cross-sections and identify the optimal *d* for each type of data and needle.

**Fig. 7:**
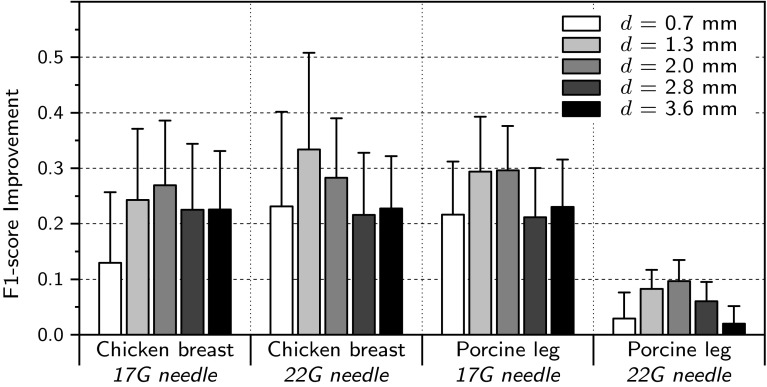
Improvements of *F*1-scores for each choice of *d*, which is the gap between the consecutive slices used as input to the three channel FCN network

Figure 7 depicts the bar chart of the measured improvement of the *F*1-scores for each dataset and choice of *d* compared to 1-channel single-slice input. The *F*1-scores are calculated after cross-validation of the predictions on parallel cross-sections to the lateral and elevational axes. As shown, adding extra consecutive cross-sections for segmentation of the needle increases the performance in all the cases. However, when the distance *d* is too large, the visible structures in the extracted cross-sections cannot be co-related to each other any longer and therefore the performance gain will decrease. As shown in Fig. 7, the spacing values of 1.3 and 2.0 mm gain the highest improvement in the *F*1-score, while the results for 2.0 mm are more stable. Therefore, we choose *d* = 2.0 mm as the optimal spacing among the consecutive cross-sections and use it in the following experiments.

#### Voxel segmentation performance

Our proposed method based on dense needle segmentation in multi-slice thick (2.5D) US planes is evaluated in terms of recall, precision and specificity, as defined in “Patch classification” section. Table 3 shows the obtained voxel-wise performances on both chicken breast and porcine leg datasets. As shown, the proposed ShareFCN architecture, achieves very high recall and precision scores in both chicken breast and porcine leg datasets.

**Fig. 8:**
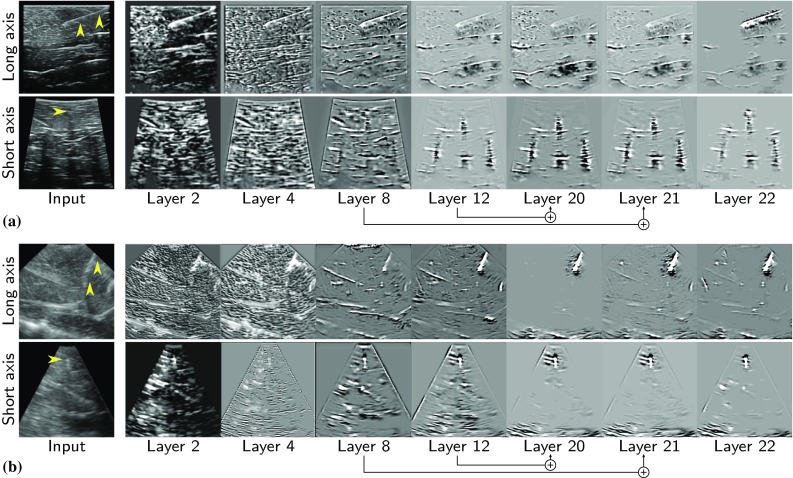
Visualization of features projected into the input space in the trained model **a** for Linear-array VL13-5 transducer and **b** for Phased-array X6-1 transducer. Reconstruction of the input image is shown by using only the highest activated features after the convolutional layers 2, 4, 8, 12, 20, 21, and 22. Note the skip connections in the layers to fuse coarse, semantic and local features. The ground-truth needle is marked with a yellow arrowhead in input images

To study the performance of our trained networks in segmenting needle voxels, we visualize the response of the intermediate feature layers to needle cross-sections. For this purpose, the reconstructed patterns from the evaluation set that cause high activations in the feature maps are visualized using the Deconvnet, as proposed by Zeiler et al. [25]. Figure 8 shows the input stimuli that creates largest excitations of individual feature maps at several layers in the model, as well as their corresponding input images. As shown, both networks trained for VL13-5 and X6-1 transducers improve the discriminating features of the needle and remove the background as the network depth increases. However, it is interesting to notice the different modeling behavior of the network in convolution layers 12, 20 and 21 for the two transducers. In the dataset acquired using the VL13-5 transducer, the higher-frequency range creates more strong shadow castings below the needle in the data. Therefore, as it can be observed in Figure 8a, the trained network additionally models the dark regions in layers 12 and 20 and fuses them to the shape and intensity features extracted in the shallower layers of the network.

**Fig. 9:**
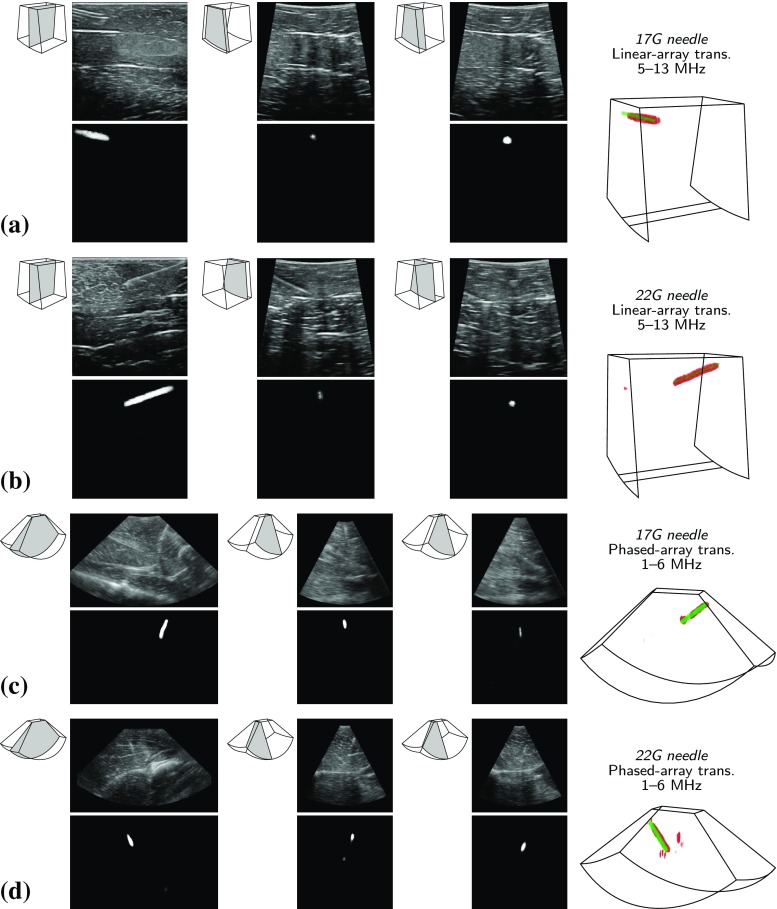
Examples of segmentation results of **a** 17 G and **b** 22 G needles in chicken breast acquired with a linear-array transducer, and **c** 17 G and **d** 22 G needles in porcine leg acquired with a phased-array transducer. Images in the top row are input cross-sections to the network and images in the bottom row are the segmentation results. Volumes on the right side show the segmented needle voxels after combining the results from both lateral and elevational directions in red and the ground-truth voxels in green

Figure 9 shows examples of the segmentation results in cross-sections perpendicular to the lateral and elevational axes. As shown, the segmentation is very accurate for all the cases of a needle being entirely visible in a cross-section, partially acquired or being viewed from the out-of-the-plane cross-sections. In particular, Fig. 9d depicts a case of a needle with a relatively large horizontal angle with the transducers, which results in the needle being partially acquired in all the processed cross-sections. As it can be seen, visible parts of the needle at each cross-section are successfully segmented and after combining the results, the needle voxels are recovered and detected in 3D.

**Table 3:** Average voxel classification performances of semantic segmentation approach in the full volumes (%)

Method	Tissue	Recall	Precision	Specificity	*F*1-score
ShareFCN$^{\hbox {a}}$	Chicken breast	89.6 ± 4.2	79.8 ± 5.5	99.97 ± 1e−4	80.0 ± 4.7
	Porcine leg	87.9 ± 4.2	83.0 ± 3.7	99.99 ± 1e−5	84.1 ± 3.4

$^{\hbox {a}}$Single model trained directly for both 17 and 22 G needles

### Axis estimation accuracy

**Fig. 10:**
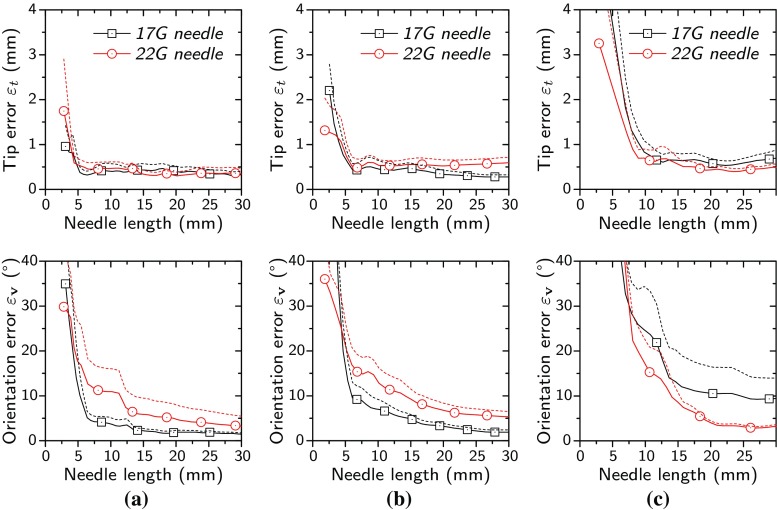
Needle tip position error ($\varepsilon _{t}$) and orientation error ($\varepsilon _{\mathbf {v}}$) as a function of needle length. Dashed lines represent standard errors of the measured values. **a** ShareCNN results in chicken breast dataset voxel size $\approx \,0.20\,\hbox {mm}$. **b** ShareFCN results in chicken breast dataset voxel size $\approx \,0.20\,\hbox {mm}$. **c** ShareFCN results in porcine leg dataset voxel size $\approx \,0.36\,\hbox {mm}$

Because of the high detection precision achieved with both ShareCNN and ShareFCN approaches, estimation of the needle axis is possible even for short needle insertions after a simple RANSAC fitting. The accuracy of our proposed ShareCNN and ShareFCN methods in localizing the needle axis is evaluated as a function of the needle length as portrayed by Fig. 10. We use two measurements for defining and evaluating spatial accuracy. The needle tip error ($\varepsilon _{t}$) is calculated as the point-plane distance of the ground-truth needle tip and the detected needle plane. The orientation error ($\varepsilon _{\mathbf {v}}$) is the angle between the detected and the ground-truth needle. As discussed earlier, we do not explicitly detect the needle tip, but detect the plane where the needle and its tip are maximally visible. This localization processing is done for every individual data point, leading to repeated detection in cases of a 3D+time US sequence.

As shown in Fig. 10a, b, both ShareCNN and ShareFCN methods perform accurately in the datasets of chicken breast, reaching $\varepsilon _\mathrm{t}$ of less than 0.7 mm for needle lengths of approximately 5 mm or larger. In both approaches, the $\varepsilon _{\mathbf {v}}$ shows more sensitivity to shorter needles and varies more for the 22 G needle, which is more difficult to estimate, compared to a thicker needle. Furthermore, for the ShareCNN method, voxels in the first 2 mm are undetectable, as the minimum distance of extracted 3D patches from the volume borders corresponds to half of a patch length.

In the datasets of porcine leg, the voxel size is reduced to 0.36 mm due to the lower acquisition frequency of the phased-array transducer. Therefore, longer lengths of the needle are required for accurate detections. As shown in Fig. 10c, for needles of approximately 10 mm or longer, the $\varepsilon _\mathrm{t}$ reduces to 0.7 and 0.6 mm for 17 and 22 G needles, respectively. In contrast to the datasets of chicken breast, the $\varepsilon _{\mathbf {v}}$ is generally larger for short 17 G needles in porcine leg. Most importantly, in all of the experiments, the needle tip error, $\varepsilon _\mathrm{t}$, remains lower than 0.7 mm. This shows that after insertion of only 5 mm for higher-resolution linear-array transducers and 10 mm for lower-resolution phased-array transducers, the tip will be always visible in the detected plane as their distance is less than the thickness of US planes.

## Discussion

From comparing the results reported in Tables 2 and 3, it can be concluded that the performance of ShareFCN is comparable and only slightly better than the patch-based ShareCNN on chicken breast data acquired from the higher-frequency range VL13-5 linear-array transducer. However, a major benefit of dense segmentation using ShareFCN is related to the data of the lower-frequency range X6-1 phased-array transducer. The resulting lower spatial resolution of these transducers distorts the appearance and obscures structure details of a needle. In these cases, training a discriminant model of the needle requires deeper and more complex convolutional networks, which increases the computational complexity. Therefore, more computationally efficient networks such as our proposed ShareFCN are preferred over patch classification methods.

Furthermore, as discussed in “Introduction” section, the US beamsteering angle of a phased-array transducer varies for each region in the field of view. Consequently, US reflections from different parts of a needle will vary largely, such that a considerable portion of the needle shaft can be virtually invisible in the data. Therefore, the receptive field of a convolutional network need to be large enough to model the contextual information from the visible parts of the needle. In a patch-based classification technique, a larger receptive field can be achieved by, e.g., increasing the patch size, increasing the number of convolution and max-pooling layers, or employing normal or atrous convolutions with larger kernel sizes. In all of these methods, the computational complexity increases exponentially as more redundant calculations have to be computed for adjacent patches, and the spatial accuracy decreases as small shifts of patches cannot be translated to two different classes.

## Conclusions

Ultrasound-guided interventions are increasingly used to minimize risks to the patient and improve health outcomes. However, the procedure of needle and transducer positioning is extremely challenging and possible external guidance tools would add to the complexity and costs of the procedure. Instead, an automated localization of the needle in 3D US can overcome 2D limitations and facilitate the ease of use of such transducers, while ensuring an accurate needle guidance. In this work, we have introduced a novel image processing system for detecting needles in 3D US data, which achieves very high precision at a low false negative rate. This high precision is achieved by exploiting dedicated convolutional networks for needle segmentation in 3D US volumes. The proposed networks are based on CNN, which is improved by proposing a new update strategy to handle highly imbalanced datasets by informed resampling of non-needle voxels. Furthermore, novel modeling of 3D US context information is introduced using 2.5D data of multi-view thick-sliced FCN.

Our proposed patch classification and semantic segmentation systems are evaluated on several ex-vivo datasets and outperform classification of the state-of-the-art handcrafted features, achieving 78 and 80% *F*1-scores in the chicken breast data, respectively. This shows the capability of CNN in modeling more semantically meaningful information in addition to simple shape features, which substantially improves needle detection in complex and noisy 3D US data. Furthermore, our proposed needle segmentation method based on 2.5D US information achieves 84% *F*1-score in datasets of porcine leg that are acquired with a lower-resolution phased-array transducer. These results show a strong semantic modeling of the needle context in challenging situations, where the intensity of the needle is inconsistent and even partly invisible.

Quantitative analysis of localization error with respect to the needle length shows that the tip error is less than 0.7 mm for needles of only 5 mm long and 10 mm long at voxel size of 0.2 and 0.36 mm, respectively. Therefore, the system is able to accurately detect short needles, enabling the physician to correct inaccurate insertions at early stages in both higher-resolution and lower-resolution datasets. Furthermore, the needle is visualized intuitively by its in-plane view while ensuring that the tip is always visible, which eliminates the need for advanced manual coordination of the transducer.

Future work will evaluate the proposed method in even more challenging in-vivo datasets with suboptimal acquisition settings. Due to the complexity of data from interventional settings, larger datasets need to be acquired for training more sophisticated networks. Moreover, further analysis is required to limit the complexity of CNN with respect to its performance for embedding this technology as a real-time application.
